# Evaluating Dietary Patterns in Women from Southern Italy and Western Mexico

**DOI:** 10.3390/nu14081603

**Published:** 2022-04-12

**Authors:** Claudia Ojeda-Granados, Martina Barchitta, Maria Clara La Rosa, Claudia La Mastra, Sonia Roman, Arturo Panduro, Antonella Agodi, Andrea Maugeri

**Affiliations:** 1 Department of Medical and Surgical Sciences and Advanced Technologies “GF Ingrassia”, University of Catania, 95123 Catania, Italy; claudiaojedagranados@hotmail.com (C.O.-G.); martina.barchitta@unict.it (M.B.); mariaclara.larosa@unict.it (M.C.L.R.); claudia.lamastra@unict.it (C.L.M.); andrea.maugeri@unict.it (A.M.); 2 Department of Genomic Medicine in Hepatology, Civil Hospital of Guadalajara “Fray Antonio Alcalde”, Hospital #278, Col. El Retiro, Guadalajara 44280, Jalisco, Mexico; soniamariaroman@hotmail.com (S.R.); apanduro@prodigy.net.mx (A.P.); 3 Health Sciences Center, University of Guadalajara, Guadalajara 44340, Jalisco, Mexico

**Keywords:** Mediterranean diet, traditional Mexican diet, food cultures, nutritional transition, women

## Abstract

Traditional diets are known to be beneficial; however, both Italian and Mexican populations are gradually moving away from the Mediterranean and traditional Mexican diets. Since women play a key role in safeguarding dietary traditions and may reflect population dietary changes, we aimed to identify Italian and Mexican women’s current dietary patterns (DPs) and characterize their nutrient content. Cross-sectional analyses were separately conducted on two convenience samples of 811 women from Southern Italy and 215 women from Western Mexico. Food frequency questionnaires, 24 h recalls, and a principal component analysis (PCA) approach were used to derive a posteriori DPs. In Italian women, the first DP was characterized by the consumption of legumes, vegetables, and fish (8.8% of the total variance), while the second DP was characterized by snack foods, processed meats, and non-olive oils. In Mexican women, the first DP was characterized by the consumption of meats and processed foods (12.6% of the total variance), while the second DP by fruits, vegetables, and whole grains. In both populations, adhering to the DPs rich in healthy foods (i.e., fruits, vegetables, legumes, and fish) was associated with a higher quality of diet in terms on nutrient content. However, adherence to the Western-type DPs was more common among women of younger age (*p* < 0.035). Thus, more extraordinary efforts are needed in promoting each country’s traditional healthy diet, especially among the new generations.

## 1. Introduction

During the last decades, one of the most important achievements of nutritional epidemiology was the design of observational studies investigating the relationships between diet and human health [1]. While earlier studies focused on the role of specific nutrients—exploiting the so-called single-nutrient approach [1]—later studies began to recognize the importance of more complex dietary patterns rich in healthy foods. As with other human behaviors, diet quality may contribute at least in part to the health inequalities observed across the globe since both dietary habits and food culture vary widely between countries.

Across the globe, the Mediterranean diet (MedDiet) and the traditional Mexican diet (TMexD) are the only ones recognized by the United Nations Educational, Scientific and Cultural Organization (UNESCO) as Intangible Cultural Heritage of Humanity [2,3]. Interestingly, this recognition also emphasized the crucial role of women in the transmission of expertise, knowledge of rituals, traditional gestures and celebrations, and the safeguarding of techniques related to these dietary patterns [2,3].

In their traditional versions, foods from the Mediterranean region and Mexico are consumed alone or as ingredients of typical dishes, constituting a culturally inherited food system. The MedDiet is generally characterized by a high consumption of cereals and other plant-based foods (vegetables, fruit, legumes, nuts, and seeds) and by the use of virgin olive oil as the main source of fat. Moreover, it is characterized by a frequent but moderate intake of red wine, moderate consumption of seafood, fermented dairy products, poultry, and eggs, and low consumption of red and processed meat and sweets [4]. However, laying down a standard definition of the MedDiet represents an ongoing task because each region in the Mediterranean Basin developed and embraced its own recipes, preferences, and restrictions [5]. For instance, Italy has been considered as a reference country for the MedDiet model [6], although some differences with other Mediterranean countries are evident. It is worth mentioning that defining the MedDiet became even more complicated due to the nutritional transition process, which began at the end of the 19th century in most countries of the Mediterranean area [7,8,9]. The TMexD, instead, is mainly characterized by grains (i.e., maize and its by-products such as tortillas), legumes, and vegetables (e.g., squash, tomato, chile, and onion). Complementary foods of the TMexD also include fruits, beverages (e.g., chocolate drinks, pulque and tesgüino fermented beverages, coffee), fish and seafood, meats, herbs and condiments, oils and fats (e.g., avocado, vegetable oils), and nuts and seeds [10]. As already stated for the MedDiet, historical changes should be evaluated when defining the TMexD, which can be considered as a blend of the pre-Hispanic Mesoamerican diet and the colonial diet after the Spanish conquest [11].

Previous studies have already suggested the beneficial effects on human health attributable to the adherence to these dietary patterns [10,12]. Yet, despite the well-known benefits, we are witnessing a progressive process of westernization of diets, which is also accompanied by rising rates of obesity and related noncommunicable diseases (NCDs) [13,14,15]. To tackle these health challenges, national government and health institutions in Italy and Mexico are showing increasing interest in identifying current dietary habits and in promoting traditional dietary patterns [16,17]. The need to identify current dietary habits has initially driven forward research on index-based methods to empirically define popular dietary patterns, including those related to the adherence to the MedDiet and to the TMexD [18,19]. However, index-based methods are based on a priori knowledge of dietary habits and/or recommendations and do not allow for identifying alternative dietary patterns in a population. To partially overcome this issue, researchers used modern exploratory data analysis approaches to identify a posteriori dietary patterns from available dietary datasets. Among these methods, principal component analysis (PCA) is commonly used to derive dietary patterns based on shared variance across dietary data within a population.

In the present study, we focused on women due to their key role in the transmission of culinary knowledge and practices. In fact, as indicated by the World Health Organization (WHO) Global Strategy on Diet, Physical Activity and Health, food and nutrition decisions are often made by women according to culture and traditional diets [20]. Moreover, a better knowledge of women’s dietary patterns could help identify opportunities to plan and implement nutrition strategies to reduce health inequalities. However, it is worth mentioning that this assumption does not apply to all countries, and hence the role of women should be assessed on a case-by-case basis. We intended to analyze the dietary habits of Italian and Mexican women using PCA to define current dietary patterns. We performed a cross-sectional analysis of dietary data from two convenience samples of adult women from Southern Italy and Western Mexico. The overall goal was to define a posteriori the dietary patterns that characterize the current women’s dietary habits and to describe their nutritional content.

## 2. Materials and Methods

### 2.1. Study Design and Populations

Cross-sectional analyses of convenience samples of adult women from Southern Italy and Western Mexico were performed. The analyses were conducted separately on each population. Italian women’s data were collected during routine physical examinations at three clinical laboratories in Catania, from 2010 to 2017, and made available by the Department of Medical and Surgical Sciences and Advanced Technologies “GF Ingrassia”, University of Catania, Italy. The Mexican women’s data were collected from the general adult population referred for medical and nutritional screening to the Nutrigenetic Clinic from 2011 to 2015, at the Department of Molecular Biology in Medicine, Civil Hospital of Guadalajara “Fray Antonio Alcalde”, Health Sciences Center, University of Guadalajara, Mexico. Age-matched (18–72 years), nonpregnant women with a complete nutritional assessment (dietary and anthropometric data) and no history of complex disease (e.g., type 2 diabetes mellitus, primary dyslipidemia, viral hepatitis, renal disease, autoimmune diseases) were selected for the analyses (*n* = 811 Italian women and *n* = 215 Mexican women). Source data studies were conducted in compliance with the Declaration of Helsinki ethical guidelines, and all women signed informed consent for data collection and analysis. The Italian study was approved by the Ethics committees “Catania 1” and “Catania 2” of the involved institutions, with the following protocol numbers: 52/2010/VE, 16/2015/CECT2, and 227/2011/BE. The Mexican study was revised and approved by the Institutional Review Board of the Health Sciences Center, University of Guadalajara, Certificate #CI-00612.

### 2.2. Dietary Assessment

In Italian women, trained epidemiologists administered a semiquantitative food frequency questionnaire (FFQ) by face-to-face interview to determine their habitual dietary intake (considering the previous month as the reference period) and derive their dietary patterns. The semiquantitative FFQ comprised 95 food items, with a twelve-choice frequency scale of consumption (from “almost never” to “two or more times a day”), and portion sizes. Medium portions were defined as the average natural sizes of unprocessed foods consumed as generally found in nature (e.g., fruits, eggs, etc.) or as the standard weight and volume of servings commonly consumed by the Italian female population. Thus, the weight of each portion depended on the type of food considered. Accordingly, small and large portions were defined as 0.5 and 1.5 times the medium portion. An indicative photographic atlas was used to support the compilation of FFQ and minimize inaccuracies, showing life-size images and the related standard portion size of all foods and drinks included in the FFQ. Medium portion sizes are also reported in the Supplementary Table S1. More details on this dietary assessment instrument are described elsewhere [21]. The dietary intake was estimated by multiplying the frequency of food consumption by the portion size (small, medium, or large), using the United States Department of Agriculture (USDA) National Nutrient Database adapted to Italian food consumption patterns, and adjusted for total energy intake by implementing the residual method [22].

The dietary assessment of Mexican women was conducted by trained dietitians through an in-person interview, as detailed elsewhere [23], using the 24 h recall (two weekdays and one weekend day) to estimate their habitual energy and nutrient intake and an FFQ to derive their dietary patterns. The 24 h recall information was processed using the NutriKal^®^ VO software (Consinfo S.C., CDMX, Mexico City, Mexico), which considers data from the USDA Nutrient Database, Release 24 [24], the food composition tables compiled by the Instituto Nacional de Ciencias Médicas y Nutrición Salvador Zubirán, the Mexican Equivalent Food System [25], as well as the nutrition facts from industrialized foods available in Mexico and the nutritional calculation of typically consumed dishes. The adjusted FFQ included 64 food items [26], with a five-choice frequency scale: daily, 1–2 times per week, ≥3 times per week, once every 15 days, and once per month. This frequency scale was transformed into a monthly consumption frequency scale for analytical purposes.

The Italian and Mexican FFQs covered foods from the traditional Mediterranean and Mexican diets, respectively, and natural or processed products commonly consumed or commercially available in each country.

### 2.3. Dietary Pattern Analysis

The PCA was performed to derive dietary patterns from women’s FFQ data as a data-driven method. For this purpose, the 95-item Italian and 64-item Mexican FFQs were reorganized into 39 and 20 food groups, respectively. It is worth mentioning that some foods were preserved as distinct groups (e.g., pizza, coffee), while others were grouped into broader groups (e.g., fruit, vegetables). Different food groups were defined according to frequency of consumption, culinary usage, and similarity of nutritional content. The orthogonal varimax rotation strategy was also applied for the PCA. The number of components (i.e., dietary patterns) to be extracted was determined by examining the scree plots and their interpretability (components showing eigenvalues ≥2.0). Then, variables (i.e., food groups) showing factor-loading scores (i.e., correlation value) ≥0.20 were considered highly correlated with dietary patterns. Accordingly, dietary patterns were named based on their representative food groups.

Adherence to the identified dietary patterns was assessed by analyzing factor scores (i.e., weighted averages of variables based on factor loadings) generated for each individual in the analysis and representing their level of adherence to each dietary pattern. Factor scores were thus ranked into tertiles (T1, T2, and T3), with T1, T2, and T3 tertile values being associated with low, medium, and high adherence, respectively. Adherence to dietary patterns was also categorized as follows: women who showed opposite tertiles (T3 vs. T1) in their adherence classification were defined as exclusively adherent to a specific dietary pattern; those showing adjacent tertiles of adherence (T1 vs. T2 or T2 vs. T2) were defined as preferable adherent to a specific dietary pattern; while those showing the same tertile (T1 vs. T1, T2 vs. T2, or T3 vs. T3) of adherence to the identified dietary patterns were defined as women with no preference for a specific dietary pattern.

### 2.4. Anthropometric Assessment

Anthropometric variables in Italian women comprised self-reported weight and height at recruitment. Anthropometric assessment in Mexican women included height, measured with a stable stadiometer (Seca GmbH & Co., KG, Hamburg, Germany), and body composition parameters (i.e., weight and body fat percentage) that were determined by bioelectrical impedance analysis using the InBody 3.0 analyzer (InBody Co., Seoul, Korea). The body mass index (BMI) was calculated as the weight in kilograms divided by the square of height in meters. Underweight (<18.5 kg/m^2^), normal weight (≥18.5–24.9 kg/m^2^), overweight (≥25.0–29.9 kg/m^2^), and obesity (≥30.0 kg/m^2^) were defined according to BMI and World Health Organization criteria.

### 2.5. Statistical Analysis

The IBM SPSS v20 software (IBM Corp., Inc., Chicago, IL, USA) was used to conduct the factor analysis and all statistical analyses, considering a *p*-value < 0.05 for statistical significance on two-sided tests. Continuous variables were expressed as median and interquartile range, while categorical variables were expressed as frequency and percentage. The normality distribution of quantitative variables was assessed with the Kolmogorov–Smirnov test with Lilliefors significance correction. Accordingly, one-way ANOVA followed by post hoc or Kruskal–Wallis test followed by Mann–Whitney test was used to compare women’s quantitative variables according to age tertiles and classifications of adherence to dietary patterns. Categorical variables were analyzed with the chi-square test.

## 3. Results

### 3.1. Characteristics of Italian and Mexican Women

The characteristics of the Italian and Mexican women groups are displayed in Table 1 and Table 2, respectively. According to BMI, the majority of Italian women (60.9%) had a normal weight, while the majority of Mexican women (75.3%) showed excess weight, also evidenced by an excess body fat percentage (>30%). We also observed that the frequency of overweight and obesity in Italian and Mexican women increased along with their age tertile (22.1%, 29.6%, 45.3% and 50.7%, 85.7%, 88.2%, respectively).

### 3.2. Dietary Patterns in Italian and Mexican Women

**Figure 1:**
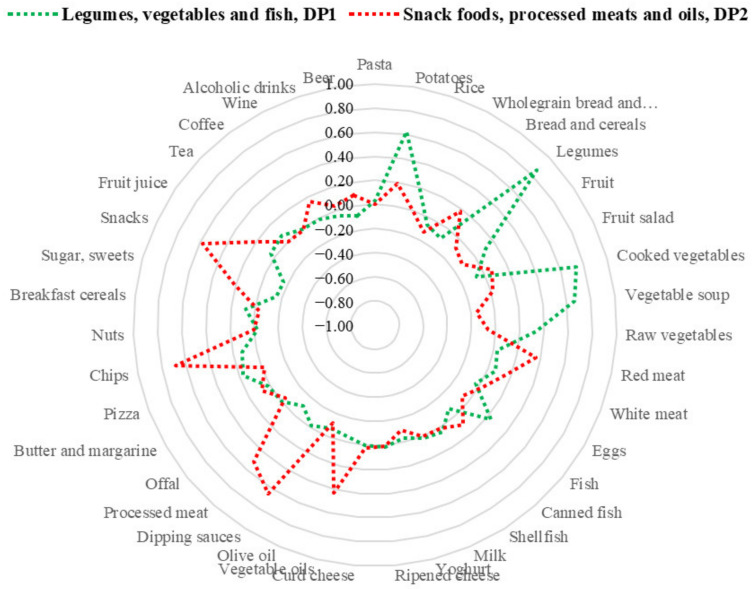
Radar plot of factor loadings of the main dietary patterns identified in Italian women.

Examination of the scree plots derived from PCA (Supplementary Figures S1 and S2) led to the identification of two main dietary patterns (i.e., components showing eigenvalues ≥2.0) in both populations. These dietary patterns explained 15.3% of the total variance among the 39 food groups considered for the Italian population and 22.8% among 20 food groups considered for the Mexican population. In both populations, the dietary patterns identified were labeled considering food groups showing factor loading scores ≥0.20 (Supplementary Tables S2 and S3). In Italian women, the first dietary pattern was named “legumes, vegetables, and fish, DP1” because it was mainly characterized by the consumption of legumes and vegetables in diverse preparations (i.e., cooked and raw vegetables, vegetable soup) and fish. The second dietary pattern was named “snack foods, processed meats, and oils, DP2”, because it was mainly characterized by the consumption of chips, dipping sauces, snacks, processed and red meat, vegetable oils, sugar, sweets, and fruit juice (Figure 1). These dietary patterns accounted for 8.8 % and 6.5% of the total variance, respectively.

**Figure 2:**
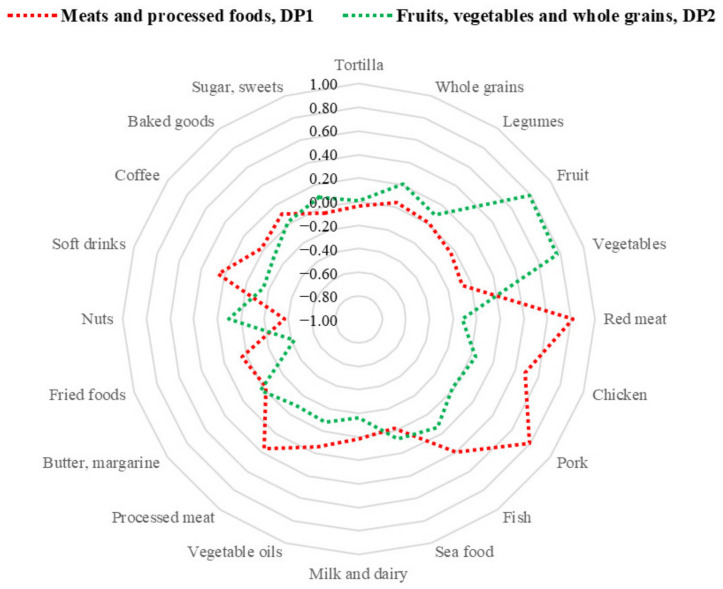
Radar plot of factor loadings of the main dietary patterns identified in Mexican women.

In Mexican women, the first dietary pattern was named “meats and processed foods, DP1” due to the high consumption of red meat, pork, chicken, fish, processed meat, and soft drinks. The second dietary pattern was named “fruits, vegetables, and whole grains, DP2” because it was mainly represented by such food groups (Figure 2). These dietary patterns explained 12.6% and 10.2% of the total variance, respectively.

### 3.3. Characteristics of Italian and Mexican Women in Relation to Adherence to Dietary Patterns

It is worth mentioning that PCA did not create mutually exclusive patterns, but rather each individual received a score of adherence to each dietary pattern derived. For this reason, we further categorized women according to the tertile distribution of factor scores. According to this classification, Table 3 and Table 4 show the distribution of women and their main characteristics. Overall, a significant difference was only observed in the age of Italian women by adherence to dietary patterns. Italian women exclusively or preferably adhering to the “snack foods, processed meats, and oils, DP2” were younger than women exclusively or preferably adhering to the “legumes, vegetables, and fish, DP1” (Table 3). Nonetheless, when comparing the age of Mexican women by adherence degree pairs, those preferably adhering to the “meats and processed foods, DP1” were younger than those exclusively adhering to the “fruits, vegetables and whole grains, DP2” (Table 4).

### 3.4. Nutritional Content according to Adherence to Italian and Mexican Dietary Patterns

Finally, we compared macro- and micronutrient intakes across adherence categories to different dietary patterns identified in Italian and Mexican women. While no differences in total energy intake were evident, the intake of some nutrients varied across the categories of adherence to different dietary patterns. In the Italian sample, women who adhered exclusively or preferably to “legumes, vegetables, and fish, DP1” exhibited higher intakes of folates, vitamin A, vitamin C, vitamin D, pyridoxine, calcium, iron, magnesium, and zinc, but lower intake of PUFAs than did those who adhered exclusively or preferably to “snacks, processed meats, and oils, DP2” (Table 5).

On the other hand, Mexican women who adhered exclusively or preferably to the “fruits, vegetables, and whole grains, DP2” showed higher intakes of folates, vitamin C, pyridoxine, and potassium but lower intake of SFA than did those who adhered exclusively or preferably to the “meats and processed foods, DP1” (Table 6).

## 4. Discussion

In this study, we sought to determine through PCA how dietary patterns differ among women from two countries that have long-standing dietary traditions. In fact, UNESCO has acknowledged the MedDiet and the TMexD as Intangible Cultural Heritage of Humanity, in which the essential role of women in conveying dietary traditions to new generations has been highlighted. Due to their cultural core and potentially beneficial impact on health and the environment, both diets are models of sustainable regional diets, with Italy, in the case of the MedDiet, being a reference country for such a model [27,28].

Our analyses, conducted separately but sharing the statistical approach, derived two distinct types of diet in both populations: a pattern characterized by the consumption of healthy foods and an alternative pattern characterized by the consumption of Western foods. The dietary pattern explaining the highest variance in dietary data among Italian women was characterized by the consumption of some foods typical of the MedDiet (i.e., legumes, vegetables, and fish). This finding partially aligned with previous studies reporting a Mediterranean-like dietary pattern as the most representative in other populations from the same country [29]. Nonetheless, it should be underlined that some differences with the traditional MedDiet were evident, such as the absence of other typical foods from this dietary pattern (e.g., fruits, cereals, and olive oil). Moreover, in Mexican women, we derived a dietary pattern mainly characterized by the consumption of healthy foods, such as fruits, vegetables, and whole grains. However, this dietary pattern essentially deviated from the TMexD, which consists of frequent consumption of maize, maize-derived foods, and legumes. Instead, it was in line with a “diverse” dietary pattern previously identified in a Mexican population, made up mostly of women [30]. Indeed, the “diverse” dietary pattern was similarly characterized by the frequent consumption of fruits, vegetables, and cereals [30].

History teaches us that, from the 1980s onwards, both Italy and Mexico have reported changes in their traditional diet and lifestyle, mainly due to economic growth and the globalization of food production [31,32]. These events led to increasing consumption of hypercaloric foods, such as those with high content of fats and sugars. Both the Italian and Mexican populations have slowly drifted away from their traditional diets, although their benefits were well recognized. This process was also accompanied by an increasing prevalence of obesity and NCDs [13,15]. As a confirmation, we derived a dietary pattern characterized by sugary and fatty Western-type foods in each population under study. Among Italian women, this dietary pattern—rich in snack foods, processed meats, and non-olive oils—was consistent with dietary patterns observed in other populations from the same region [21,33,34]. Among Mexican women, the Western-like dietary pattern was mainly characterized by the consumption of meats and processed foods, and it explained the highest variance in dietary data. This result differed from what was observed among Italian women, in which the Mediterranean-like dietary pattern was the most representative. There are some explanations for this difference. Firstly, it has been supposed that more drastic changes occurred in Mexico than Italy in the pronounced availability and consumption of ultra-processed products while neglecting traditional foods [13,35,36]. This behavior probably contributed to the fact that the Western-type dietary pattern is now the most common among Mexican women and the general population. Secondly, the difference in the most representative dietary pattern could reflect the different distribution of overweight and obesity between the two countries. While most Italian women had a normal body weight, most Mexican women were overweight or obese. Nevertheless, we found no differences in BMI and the proportion of overweight and obesity by categories of adherence to dietary patterns, neither in Italian nor in Mexican women. In this regard, changes in the consumption of some foods have not been uniform over time in Italy; while some have increased, others have decreased, thus balancing diets in terms of energy supply [13,37]. For example, during the last twenty years, there has been a decline in the consumption of beef, animal fats, milk, dairy products, and, more recently, vegetables and fruits, while the consumption of nuts and tropical oils has increased. In the case of Mexico, there has been a considerable increase in the consumption of sugar, vegetable oils, animal fat, and foods of animal origin (e.g., beef, pork, poultry, eggs), while that of maize, cereals, legumes, fruit, and vegetables has dropped [35]. Moreover, both the Western-type and the “diverse” dietary patterns previously identified in the general population were associated with a higher risk of overweight or obesity if compared with a traditional dietary pattern, which included Mexican staple foods such as maize and its derivatives [30].

In line with these findings, no difference in the total energy intake of Italian and Mexican women was observed by categories of adherence to dietary patterns. By contrast, the intake of some vitamins and minerals significantly varied. As expected, dietary patterns rich in healthy foods were also characterized by healthier nutritional content. In particular, adhering to the Italian “legumes, vegetables, and fish DP1” was significantly associated with a higher intake of folates, vitamin A, vitamin C, vitamin D, pyridoxine, calcium, iron, magnesium, and zinc, but lower intake of PUFAs. Similarly, adhering to the Mexican “fruits, vegetables, and whole grains, DP2” was associated with higher intake of folates, vitamin C, pyridoxine, and potassium, but a lower intake of SFAs. Nonetheless, the intakes of vitamin D and calcium in Italian women and folates, pyridoxine, and potassium in Mexican women were still below the general Recommended Dietary Allowances values [38]. Altogether, these findings confirmed previous evidence that traditional dietary patterns—especially those rich in plant-based foods—are likely to be of higher quality in terms of micronutrients beneficial to health [30,39]. Among these, folates play an essential role for women during the periconceptional period, pregnancy, and in offspring’s short- and long-term outcomes [40]. Regarding this issue, it is worth mentioning that the Italian and Mexican populations present a high frequency of the T allele of the *MTHFR* C677T polymorphism [41], which, if combined with insufficient folate intake, makes them more prone to adverse outcomes during different stages of life [42,43]. This is another reason to promote the adherence to traditional and healthy dietary patterns in these populations.

As reported by previous studies, we also showed a relationship between age and diet quality [22]. In particular, women adhering to the Mediterranean-like dietary pattern were older than those adhering to the Western-type dietary pattern. It has been noted that younger generations are prone to abandon the traditional MedDiet by adopting unhealthy dietary trends [44,45]. The same has been documented for older Mexican adults, especially women, who consume a diet of higher quality and traditional type compared with the diet of younger generations [36,46]. Therefore, it is likely that older adults and particularly women in Italy and Mexico, still preserve more dietary traditions than younger people do.

Our study has several strengths. Firstly, it demonstrated that PCA could be applied to derive current dietary patterns in countries with a rooted dietary tradition. This technique could also provide evidence for potential changes from traditional diets. Secondly, our analyses were conducted on large cohorts of women from the populations of Mexico and Italy. These analyses were performed separately but shared the same statistical approach. Thirdly, data collection was performed through standard and validated tools that were almost consistent between the two cohorts. Finally, dietary patterns were derived through PCA, and their interpretation was consistent with that of previous studies in similar populations. Taken together, these strengths allowed us to detect dietary patterns in populations with a strong food culture and to monitor how they are drifting from traditional regional diets to a more westernized diet. Moreover, our approach was also helpful in revealing the age groups where the consumption of non-endemic food is taking place. These findings can aid in implementing age-specific strategies to combat the lack of awareness of unhealthy eating habits.

Our study, however, also had some limitations. Firstly, its cross-sectional design does not allow the assessment of causality. Moreover, our work cannot be considered a comparative study because data were analyzed separately for each population. Secondly, we worked on age-matched convenience samples of women, which did not represent the entire countries. Thirdly, the dietary assessment was performed using instruments that did not preclude potential measurement errors and may suffer from inaccuracies. For example, the FFQ used for the Italian population was based on standard portion sizes defined a priori. However, to reduce the degree of inaccuracy, participants were supported by a photographic atlas to fill out the questionnaire. Similarly, data on the weight and height of the Italian population were self-reported, thus suffering from inaccuracies compared to objectively collected information. Moreover, instruments used for dietary assessment differed between the Italian and Mexican samples, precluding a proper comparison between countries. With respect to this limitation, it would have been appropriate to analyze biochemical values to assess the effect of adherence to the dietary patterns identified. Thirdly, we did not evaluate the effects of several unmeasured factors that could influence the dietary habits of recruited women, such as those related to the social sphere (e.g., income, food security, food access). Regarding this issue, it should be noted that we analyzed two countries in a different phase of economic and human development: Italy, a fully developed country with a per capita gross domestic product (GDP) of USD 43,000 in 2021; Mexico, a country still developing with a per capita GDP of USD 22,000.

## 5. Conclusions

Our study confirms that PCA is a valid approach to evaluate dietary patterns in countries where traditional diets exist. It was also possible to determine whether current dietary habits deviate from the MedDiet and from the TMexD. We identified a healthy dietary pattern and a Western dietary pattern in both populations. However, while the healthy dietary pattern of Italian women included various typical MedDiet foods, that of Mexican women lacked several TMexD staples, such as maize, maize-derived foods, and beans. Nevertheless, both dietary patterns proved to be of higher quality—in terms of micronutrients beneficial to health—compared with Western dietary patterns. Unfortunately, a relationship between women’s younger age and adherence to Western-type dietary patterns was observed. Therefore, more extraordinary efforts and actions are required to promote women’s dietary traditions, especially for new generations.

## Figures and Tables

**Table 1:** Characteristics of Italian women.

Characteristic	Italian Women (*n* = 811)	Age Tertile			*p*-Value
		18–33 Years	34–46 Years	47–72 Years	
Age, years	40.0 (19)	28.0 (6.0)	40.0 (6)	54.0 (11)	<0.001
Weight, kg	60.0 (15.0)	57.0 (15.0)	60.0 (15.0)	63.0 (13.0)	<0.001
BMI, kg/m^2^	22.9 (5.2)	21.2 (5.0)	22.9 (4.8)	24.2 (5.1)	<0.001
Underweight, *n* (%)	56 (7.0)	36 (13.7)	16 (5.6)	4 (1.6)	<0.001
Normal weight, *n* (%)	490 (60.9)	168 (64.1)	186 (64.8)	136 (53.1)	
Overweight, *n* (%)	175 (21.7)	37 (14.1)	56 (19.5)	82 (32.0)	
Obesity, *n* (%)	84 (10.4)	21 (8.1)	29 (10.1)	34 (13.3)	

Variables are reported as median and (interquartile range) or frequency and (percentage). BMI, body mass index. The Kruskal–Wallis test was used to analyze continuous variables due to the non-normal distribution of data. Categorical variables were analyzed with the chi-square test.

**Table 2:** Characteristics of Mexican women.

Characteristic	Mexican Women (*n* = 215)	Age Tertile			*p*-Value
		18–31 Years	32–45 Years	46–72 Years	
Age, years	40.0 (21)	23.0 (7.0)	40.0 (5)	53.0 (11)	<0.001
Weight, kg	72.0 (21.0)	66.0 (23.0) ^a^	76.5 (20.0)	74.0 (19.8)	0.005
Body fat, %	36.8 (10.5)	32.7 (12.5) ^b^	38.3 (7.0)	38.3 (9.5)	<0.001
BMI, kg/m^2^	29.4 (8.8)	25.5 (8.9) ^c^	30.2 (6.5)	31.0 (8.1)	<0.001
Normal weight, *n* (%)	53 (24.7)	34 (49.3)	10 (14.3)	9 (11.8)	<0.001
Overweight, *n* (%)	60 (27.9)	17 (24.6)	21 (30.0)	22 (28.9)	
Obesity, *n* (%)	102 (47.4)	18 (26.1)	39 (55.7)	45 (59.3)	

Variables are reported as median and (interquartile range) or frequency and (percentage). BMI, body mass index. The one-way ANOVA test was used to analyze continuous variables due to normally distributed data. Bonferroni post hoc test: ^a^ 1st age tertile vs. 2nd and 3rd, *p* < 0.020. Dunnette’s T3 post hoc test: ^b^ 1st age tertile vs. 2nd and 3rd, *p* < 0.002; ^c^ 1st age tertile vs. 2nd and 3rd, *p* < 0.001. Categorical variables were analyzed with the chi-square test.

**Table 3:** Characteristics of 811 Italian women by adherence to dietary patterns.

Characteristic	Adherence to Dietary Patterns					*p*-Value
	Exclusively “Snack Foods, Processed Meats, and Oils, DP2” (*n* = 85)	Preferably “Snack Foods, Processed Meats, and Oils, DP2” (*n* = 178)	No Preference (*n* = 285)	Preferably “Legumes, Vegetables, and Fish, DP1” (*n* = 178)	Exclusively “Legumes, Vegetables, and Fish, DP1” (*n* = 85)	
Age, years	38.0 (14.0) ^a^	37.0 (17.0) ^b^	40.0 (20.0)	42.0 (21.0)	42.0 (18.0)	0.001
Weight, kg	58.0 (11.8)	60.0 (15.8)	60.0 (16.0)	62.0 (13.5)	62.5 (15.0)	0.065
BMI, kg/m^2^	22.1 (5.5)	22.6 (4.9)	23.1 (6.2)	23.0 (5.0)	23.1 (6.2)	0.273
Underweight, *n* (%)	8 (14.3)	16 (28.6)	20 (35.7)	8 (14.3)	4 (4.8)	0.933
Normal weight, *n* (%)	51 (10.4)	105 (21.4)	171 (34.9)	113 (23.1)	50 (10.2)	
Overweight, *n* (%)	18 (10.3)	38 (21.7)	62 (35.4)	36 (20.6)	21 (12.0)	
Obesity, *n* (%)	84 (10.4)	176 (21.9)	285 (35.4)	176 (21.9)	84 (10.4)	

Variables are reported as median and (interquartile range) or frequency and (percentage). DP, dietary pattern; BMI, body mass index. The Kruskal–Wallis test was used to analyze continuous variables due to the non-normal distribution of data. Mann–Whitney test: ^a^ exclusively DP2 vs. exclusively DP1 and preferably DP1, *p* = 0.006 and *p* = 0.046; ^b^ preferably DP2 vs. exclusively DP1, preferably DP1, and no preference, *p* < 0.001, *p* = 0.002, *p* = 0.015. Categorical variables were analyzed with the chi-square test.

**Table 4:** Characteristics of 215 Mexican women by adherence to dietary patterns.

Characteristic	Adherence to Dietary Patterns					*p*-Value
	Exclusively “Meats and Processed Foods, DP1” (*n* = 26)	Preferably “Meats and Processed Foods, DP1” (*n* = 40)	No Preference (*n* = 80)	Preferably “Fruits, Vegetables, and Whole Grains, DP2” (*n* = 46)	Exclusively “Fruits, Vegetables, and Whole Grains, DP2” (*n* = 23)	
Age, years	41.0 (21.0)	37.5 (22.0) ^a^	41.0 (21.0)	40.0 (21.0)	45.0 (29.0)	0.109
Weight, kg	77.0 (31.3)	75.1 (17.8)	72.5 (21.3)	71.5 (18.8)	68.0 (15.0)	0.363
Body fat, %	38.5 (16.9)	38.0 (8.8)	37.5 (9.7)	34.8 (9.4)	35.6 (8.8)	0.186
BMI, kg/m^2^	32.1 (12.6)	29.5 (7.9)	29.9 (9.5)	28.7 (7.5)	26.7 (6.8)	0.461
Normal weight, *n* (%)	8 (15.4)	8 (15.4)	16 (30.8)	12 (23.1)	8 (15.4)	0.396
Overweight, *n* (%)	3 (5.0)	12 (20.0)	21 (35.0)	16 (26.7)	8 (13.3)	
Obesity, *n* (%)	15 (15.5)	20 (20.6)	37 (38.1)	18 (18.6)	7 (7.2)	

Variables are reported as median and (interquartile range) or frequency and (percentage). DP, dietary pattern; BMI, body mass index. The one-way ANOVA test was used to analyze continuous variables due to normally distributed data. ^a^ preferably DP1 vs. exclusively DP2, *p* = 0.017 (*t*-test). Categorical variables were analyzed with the chi-square test.

**Table 5:** Macro- and micronutrient intake of 811 Italian women by adherence to dietary patterns.

Nutrients	Adherence to Italian Dietary Patterns					*p*-Value
	Exclusively “Snack Foods, Processed Meats, and Oils, DP2” (*n* = 85)	Preferably “Snack Foods, Processed Meats, and Oils, DP2” (*n* = 178)	No Preference (*n* = 285)	Preferably “Legumes, Vegetables, and Fish, DP1” (*n* = 178)	Exclusively “Legumes, Vegetables, and Fish, DP1” (*n* = 85)	
Total energy, kcal	2015.4 (634.0)	1878.9 (639.0)	1916.2 (805.2)	1940.8 (661.1)	1942.1 (548.6)	0.719
SFAs, %	25.4 (10.2)	23.7 (13.2)	23.0 (11.7)	23.7 (10.6)	22.6 (8.9)	0.161
MUFAs, %	44.9 (19.8)	43.4 (23.9)	43.9 (23.1)	45.1 (22.5)	39.8 (22.2)	0.675
PUFAs, %	14.5 (6.5)	13.5 (6.2)	12.8 (5.3)	12.7 (4.5) ^a^	12.4 (5.0) ^a^	0.001
Folates, µg/d DFEs	184.7 (99.0)	230.1 (112.0)	264.7 (153.2)	319.3 (172.5) ^b^	404.6 (146.1) ^b^	<0.001
Vitamin A, µg/d	762.9 (471.6)	889.7 (558.3)	1057.8 (849.1)	1276.3 (911.6) ^c^	1675.0 (944.0) ^c^	<0.001
Vitamin C, mg/d	95.2 (96.8)	88.7 (98.3)	109.5 (122.3)	132.6 (139.9) ^d^	149.3 (161.3) ^d^	<0.001
Vitamin D, µg/d	3.8 (3.9)	3.8 (3.3)	4.3 (5.5)	4.9 (5.6) ^e^	7.4 (5.5) ^f^	<0.001
Thiamin, mg/d	1.4 (0.6)	1.4 (0.6)	1.5 (0.7)	1.5 (0.6)	1.5 (0.5)	0.054
Pyridoxine, mg/d	1.9 (0.7)	1.9 (0.9)	1.9 (1.0)	2.0 (1.0) ^g^	2.3 (0.8) ^h^	<0.001
Calcium, mg/d	743.9 (385.1)	816.1 (444.6)	825.6 (503.2)	872.3 (384.5) ^i^	930.9 (416.9) ^i^	0.007
Iron, mg/d	11.2 (4.8)	12.0 (5.9)	12.8 (7.7)	13.8 (6.9) ^j^	14.6 (6.5) ^j^	<0.001
Magnesium, mg/d	262.7 (97.0)	284.0 (99.0)	296.4 (137.7)	307.8 (122.0) ^k^	346.3 (98.5) ^l^	<0.001
Zinc, mg/d	8.8 (3.7)	8.6 (3.6)	8.7 (4.5)	8.9 (3.4)	9.5 (3.0) ^m^	0.039

Variables are reported as median and (interquartile range). DP, dietary pattern; SFAs, saturated fatty acids; MUFAs, monounsaturated fatty acids; PUFAs, polyunsaturated fatty acids; DFEs, dietary folate equivalents; d, day. Statistical analysis was performed using the Kruskal–Wallis test followed by Mann–Whitney test when pertinent. ^a^ Exclusively DP1 or preferably DP1 vs. preferably DP2 and exclusively DP2 (*p* < 0.032); ^b^ exclusively DP1 or preferably DP1 vs. all (*p* < 0.001); ^c^ exclusively DP1 or preferably DP1 vs. all (*p* < 0.012); ^d^ exclusively DP1 or preferably DP1 vs. preferably DP2, exclusively DP2 and no preference (*p* < 0.025); ^e^ preferably DP1 vs. preferably DP2 (*p* = 0.007); ^f^ exclusively DP1 vs. all (*p* < 0.004); ^g^ preferably DP1 vs. preferably DP2 (*p* = 0.028); ^h^ exclusively DP1 vs. all (*p* < 0.002); ^i^ exclusively DP1 or preferably DP1 vs. exclusively DP2 (*p* < 0.016); ^j^ exclusively DP1 or preferably DP1 vs. preferably DP2 and exclusively DP2 (*p* < 0.003); ^k^ preferably DP1 vs. preferably DP2 and exclusively DP2 (*p* = 0.001); ^l^ exclusively DP1 vs. all (*p* < 0.001); ^m^ exclusively DP1 vs. all (*p* < 0.015).

**Table 6:** Macro- and micronutrient intake of 215 Mexican women by adherence to dietary patterns.

Nutrients	Adherence to Mexican Dietary Patterns					*p*-Value
	Exclusively “Meats and Processed Foods, DP1” (*n* = 26)	Preferably “Meats and Processed Foods, DP1” (*n* = 40)	No Preference (*n* = 80)	Preferably “Fruits, Vegetables, and Whole Grains, DP2” (*n* = 46)	Exclusively “Fruits, Vegetables, and Whole Grains, DP2” (*n* = 23)	
Total energy, kcal	1866.5 (1001.5)	1804.5 (635.5)	1896.5 (806.5)	1754.0 (555.5)	1717.0 (518.0)	0.731
Protein, %	17.5 (9.0)	16.0 (4.3)	17.0 (6.0)	17.0 (4.3)	17.0 (7.0)	0.828
Total fat, %	36.0 (12.3)	33.0 (11.0)	34.0 (12.8)	30.0 (14.5)	31.0 (20.0)	0.371
SFAs, %	10.0 (6.0)	10.0 (5.3)	10.0 (6.0)	9.0 (5.5)	6.0 (6.0) ^a^	0.013
MUFAs, %	11.0 (7.3)	10.0 (5.5)	10.0 (6.8)	9.5 (6.3)	9.0 (11.0)	0.980
PUFAs, %	5.0 (4.8)	4.0 (3.0)	4.0 (2.0)	5.0 (4.0)	4.0 (5.0)	0.894
Cholesterol, mg	308.5 (299.8)	245.0 (195.8)	211.0 (836.0)	207.5 (148.3)	260.0 (249.0)	0.557
Carbohydrates, %	49.0 (9.8)	52.0 (11.3)	51.5 (15.0)	53.5 (12.5)	54.0 (13.0)	0.407
Fiber, g/d	15.5 (15.3)	18.0 (15.0)	18.0 (18.0)	19.0 (18.0)	20.0 (17.0)	0.408
Folates, µg/d DFEs	114.9 (113.6)	85.2 (70.4)	148.1 (133.3)	177.0 (149.8) ^b^	238.9 (248.8) ^b^	<0.001
Vitamin A, µg/d	585.0 (1374.0)	364.5 (910.0)	733.0 (1349.0)	665.0 (1082.0)	840.0 (1602.0)	0.087
Vitamin C, mg/d	41.2 (117.1)	38.6 (97.5)	55.2 (100.2)	94.0 (109.7) ^c^	158.8 (232.5) ^c^	0.002
Vitamin E, mg/d	2.2 (3.2)	1.4 (1.8)	2.2 (3.4)	2.1 (2.6)	2.4 (2.0)	0.363
Thiamin, mg/d	1.1 (0.7)	0.9 (0.7)	1.2 (0.9)	1.2 (0.6)	1.1 (0.8)	0.038
Riboflavin, mg/d	1.2 (0.7)	1.0 (0.7)	1.3 (1.0)	1.4 (0.9)	1.2 (0.8)	0.181
Niacin, mg/d	18.5 (17.5)	11.2 (11.7)	14.5 (12.9)	15.8 (12.4)	15.6 (9.3)	0.424
Pyridoxine, mg/d	1.1 (0.9)	1.1 (1.1)	1.1 (0.9)	1.6 (0.9) ^d^	1.4 (1.4)	0.006
Cobalamin, µg/d	2.5 (4.4)	2.4 (1.8)	2.4 (2.2)	2.3 (3.1)	1.7 (1.9)	0.531
Pantothenic acid, mg/d	1.8 (1.2)	1.7 (1.5)	2.1 (1.6)	2.0 (1.6)	2.4 (1.9)	0.487
Calcium, mg/d	959.5 (732.0)	889.5 (501.0)	938.0 (543.0)	869.5 (460.0)	932.0 (764.0)	0.646
Iron, mg/d	11.3 (10.2)	12.3 (6.3)	14.2 (9.9)	13.9 (10.6)	13.6 (7.0)	0.442
Sodium, g/d	1.9 (2.0)	2.0 (1.1)	2.1 (1.7)	1.7 (1.3)	1.5 (1.4)	0.146
Potassium, mg/d	1576.5 (990.0)	1707.5 (869.0)	1966.0 (1199.0)	2351.0 (1207.0) ^e^	2352.0 (1580.0) ^f^	0.003
Selenium, µg/d	41.0 (45.0)	36.0 (27.0)	45.0 (41.0)	37.5 (39.0)	41.0 (33.0)	0.286
Phosphorus, mg/d	583.0 (473.0)	620.0 (412.0)	737.0 (451.0)	705.0 (485.0)	690.0 (641.0)	0.781
Magnesium, mg/d	171.5 (285.0)	185.5 (233.0)	224.0 (273.0)	258.5 (186.0)	244.0 (182.0)	0.377
Zinc, mg/d	6.2 (6.9)	6.2 (5.7)	6.5 (4.2)	6.4 (5.2)	5.9 (3.6)	0.615

Variables are reported as median and (interquartile range). DP, dietary pattern; SFAs, saturated fatty acids; MUFAs, monounsaturated fatty acids; PUFAs, polyunsaturated fatty acids; DFEs, dietary folate equivalents; d, day. When pertinent, statistical analysis was performed using the Kruskal–Wallis test followed by the Mann–Whitney test. ^a^ Exclusively DP2 vs. preferably DP1, exclusively DP1 and no preference (*p* < 0.035); ^b^ exclusively DP2 or preferably DP2 vs. preferably DP1 and exclusively DP1 (*p* < 0.050); ^c^ exclusively DP2 or preferably DP2 vs. preferably DP1, exclusively DP1, and no preference (*p* < 0.032); ^d^ preferably DP2 vs. preferably DP1, exclusively DP1 and no preference (*p* < 0.006); ^e^ preferably DP2 vs. preferably DP1, exclusively DP1, and no preference (*p* < 0.010); ^f^ exclusively DP2 vs. preferably DP1 (*p* < 0.037).

## Data Availability

The data presented in this study are available on request from the corresponding author.

## Supplementary Materials

The following supporting information can be downloaded at: https://www.mdpi.com/article/10.3390/nu14081603/s1, Table S1: Medium or standard portion size of foods included in the food frequency questionnaire used for Italian women; Figure S1: Scree plot of the eigenvalues of components extracted from Italian women’s food frequency questionnaire data; Figure S2: Scree plot of the eigenvalues of components extracted from Mexican women’s food frequency questionnaire data; Table S2: Factor loading matrix of main dietary patterns identified in Italian women; Table S3: Factor loading matrix of main dietary patterns identified in Mexican women.
