# Supplementary material for: Evaluating Dietary Patterns in Women from Southern Italy and Western Mexico
Source: Nutrients. 2022 Apr 12;14(8):1603. doi: 10.3390/nu14081603 (PMC9027885; doi:10.3390/nu14081603)
Supplement: Supplementary file 1 [file nutrients-14-01603-s001.zip › nutrients-1660579-supplementary.pdf]

Supplementary Material

Table S1. Medium or standard portion size of foods included in the food frequency questionnaire used for Italian women.

95 Food Items	Portion (grams)
Apple	150
Apricot	120
Artichoke	150
Aubergine	150
Banana	110
Beef, veal, lamb meat	120
Boiled potatoes	150
Bresaola	70
Brioches	80
Butter and margarine	20
Cake, pastries	70
Candy	20
Canned fish	100
Carrots	100
Cauliflower, broccoli	80
Cereals	30
Cherries	150
Chocolate, snack chocolate	30
Cooked vegetables	150
Cookies	30
Eggs	120
Fennel	150
Fesa, chicken breast	40
Fish (high in fat)	150
Fish (low in fat)	150
Fish (medium in fat)	150
Fresh tomatoes	150
Fries	150
Fruit salad	150
Full fat yoghurt	125
Green bean	150
Green salad	100
Ham (cooked or uncooked)	40
Hard cheeses	50
Horse meat	120
Ice cream	100
Jam, marmalade	10
Ketchup	10
Kiwi	150
Legumes	120
Low fat yoghurt	125
Mayonnaise	10

Melon, mango	150
Mortadella	40
Mushroom	150
Nuts	15
Offal	60
Olive oil	30
Orange, mandarin	150
Pasta	60
Pastry cream, pudding	30
Peach, nectarine, prune	150
Pear	150
Peas	125
Peppers	150
Pineapple	100
Pizza	150
Popcorn, pretzels	15
Pork meat	120
Red grape	150
Rice	70
Rusks, crackers	30
Rusks, wholemeal crackers	30
Salami	30
Shellfish	100
Soft cheeses	120
Soup	150
Spinach	150
Strawberries	150
Vegetable oil	30
Watermelon	200
White grape	150
White meat	100
Wholemeal biscuits	30
Wholemeal bread	100
Wrapped potato chips	25
Wurstel chicken	50
Wurstel pork	50
Zucchini, pumpkin	150
Spirits and other alcoholic drinks	
Beer	
Coffee	
Multivitamin juice	
Pineapple juice	
Orange juice	Portion sizes were indicated through a photographic atlas showing different life-size cups and glasses
Pear juice	
Peach juice	
Whole milk	
Semi-skimmed milk	
Skimmed milk	

Tea
Energy drinks
Red wine
Rosè wine, white wine
Water

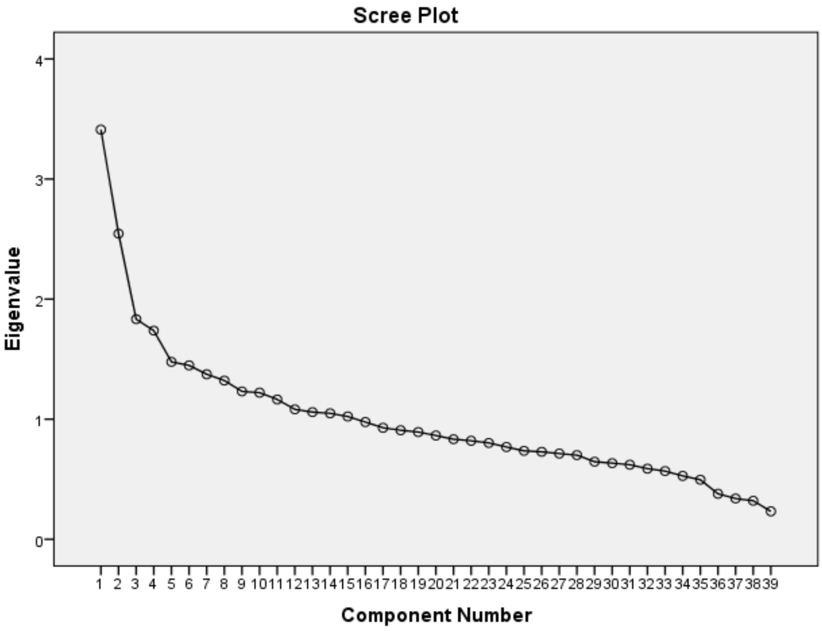

Figure S1. Scree plot of the eigenvalues of components extracted from Italian women's food frequency questionnaire data.

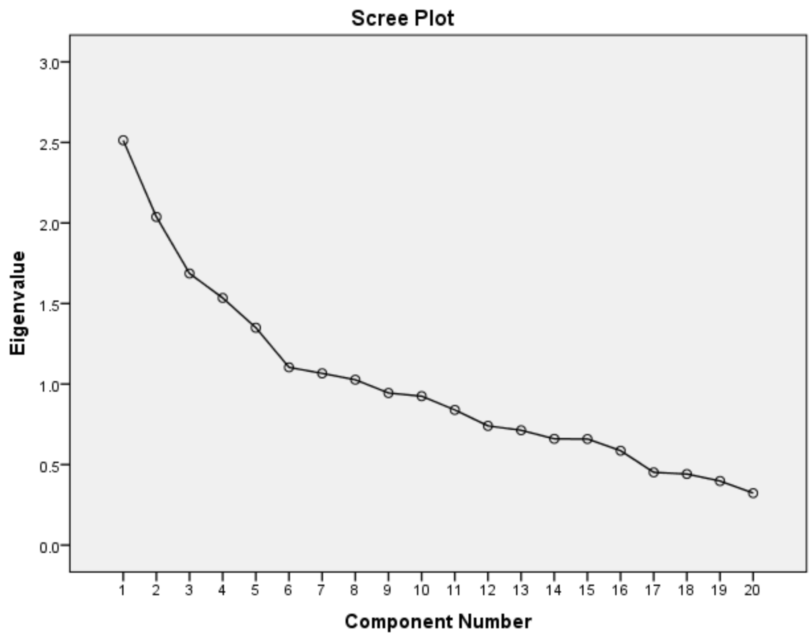

Figure S2. Scree plot of the eigenvalues of components extracted from Mexican women's food frequency questionnaire data.

Table S2. Factor loading matrix of main dietary patterns identified in Italian women.

Food groups	Dietary patterns	
	**Legumes, vegetables and fish, DP1**	**Snack foods, processed meats and oils, DP2**
Pasta	0.030	0.001
Potatoes	**0.622**	0.188
Rice	0.182	-0.002
Wholegrain bread and cereals	-0.062	-0.130
Bread and cereals	-0.097	0.178
Legumes	**0.860**	-0.075
Fruit	0.112	-0.118
Fruit salad	-0.065	0.074
Cooked vegetables	**0.734**	0.012
Vegetable soup	**0.665**	-0.156
Raw vegetables	**0.330**	-0.058
Red meat	0.030	**0.358**
White meat	0.065	0.159
Eggs	-0.033	0.028
Fish	**0.238**	-0.066
Canned fish	-0.077	0.103
Shellfish	0.047	0.016
Milk	0.019	0.003
Yoghurt	-0.035	-0.103
Ripened cheese	0.015	0.007
Curd cheese	0.003	0.020

Vegetable oils	-0.049	**0.436**
Olive oil	-0.068	-0.121
Dipping sauces	-0.018	**0.655**
Processed meat	-0.109	**0.513**
Offal	-0.003	-0.041
Butter and margarine	0.016	0.083
Pizza	0.168	-0.021
Chips	0.124	**0.679**
Nuts	-0.026	0.003
Breakfast cereals	0.076	-0.032
Sugar, sweets	-0.148	**0.216**
Snacks	-0.166	**0.576**
Fruit juice	0.043	**0.210**
Tea	0.068	-0.001
Coffee	-0.005	-0.011
Wine	-0.012	0.157
Alcoholic drinks	-0.043	0.042
Beer	-0.085	0.090

Food groups representative of dietary patterns according to a factor loading value of ≥ 0.20 are in bold. DP, dietary pattern.

Table S3. Factor loading matrix of main dietary patterns identified in Mexican women.

**Food groups**	**Dietary patterns**	
	**Meats and processed foods, DP1**	**Fruits, vegetables and whole grains, DP2**
Tortilla	-0.037	0.003
Whole grains	0.039	**0.208**
Legumes	0.011	0.094
Fuits	-0.037	**0.788**
Vegetables	-0.074	**0.767**
Red meat	**0.815**	-0.122
Chicken	**0.486**	0.043
Pork	**0.790**	-0.014
Fish	**0.398**	0.137
Sea food	-0.026	0.072
Milk and dairy	0.019	-0.163
Vegetable oils	0.142	-0.082
Processed meat	**0.358**	-0.090
Butter, margarine	-0.026	0.031
Fried foods	0.037	-0.424
Nuts	-0.370	0.103
Soft drinks	**0.250**	-0.147
Coffee	0.021	-0.107
Baked goods	0.105	0.018
Sugar, sweets	-0.053	0.092

Food groups representative of dietary patterns according to a factor loading value of ≥ 0.20 are in bold. DP, dietary pattern.
